# A *Drosophila* Toolkit for the Visualization and Quantification of Viral Replication Launched from Transgenic Genomes

**DOI:** 10.1371/journal.pone.0112092

**Published:** 2014-11-11

**Authors:** Mathias F. Wernet, Martha Klovstad, Thomas R. Clandinin

**Affiliations:** Department of Neurobiology, Stanford University School of Medicine, Stanford, California, United States of America; Indiana University, United States of America

## Abstract

Arthropod RNA viruses pose a serious threat to human health, yet many aspects of their replication cycle remain incompletely understood. Here we describe a versatile *Drosophila* toolkit of transgenic, self-replicating genomes (‘replicons’) from Sindbis virus that allow rapid visualization and quantification of viral replication *in vivo*. We generated replicons expressing Luciferase for the quantification of viral replication, serving as useful new tools for large-scale genetic screens for identifying cellular pathways that influence viral replication. We also present a new binary system in which replication-deficient viral genomes can be activated ‘in trans’, through co-expression of an intact replicon contributing an RNA-dependent RNA polymerase. The utility of this toolkit for studying virus biology is demonstrated by the observation of stochastic exclusion between replicons expressing different fluorescent proteins, when co-expressed under control of the same cellular promoter. This process is analogous to ‘superinfection exclusion’ between virus particles in cell culture, a process that is incompletely understood. We show that viral polymerases strongly prefer to replicate the genome that encoded them, and that almost invariably only a single virus genome is stochastically chosen for replication in each cell. Our *in vivo* system now makes this process amenable to detailed genetic dissection. Thus, this toolkit allows the cell-type specific, quantitative study of viral replication in a genetic model organism, opening new avenues for molecular, genetic and pharmacological dissection of virus biology and tool development.

## Introduction

Arboviruses like Dengue, Yellow Fever Virus, West Nile Virus, and tick-borne encephalitis virus are spread by arthropod hosts and infect millions of patients per year, with neither effective vaccines, nor specific antiviral therapies [1]–[3]. The related alphavirus Sindbis serves as a powerful model for studying RNA virus biology [4]–[5]. Sindbis displays a wide host range, from mammals to insects [6], including *Drosophila* [7]. The dissection of the Sindbis life cycle has long focused on mammalian cell-culture systems using both purified virus particles or self-replicating genomes incapable of forming virus particles (‘replicons’) [8], [9]. Significantly less was known about virus replication in the insect host. The recent introduction of genetically-inducible, self-amplifying Sindbis replicons that are stably inserted into the *Drosophila* genome promise an even more detailed study of host factors affecting viral transcription and replication *in vivo* [10]–[13]. We have recently shown that infectious Sindbis particles can be produced *in vivo*, in a cell-type specific manner, through trans-complementation from inducible, transgenic replicons [13]. Here we develop an extended toolkit of transgenic replicons for the rapid visualization and quantitative study of Sindbis replication in this insect host.

**Figure 1:**
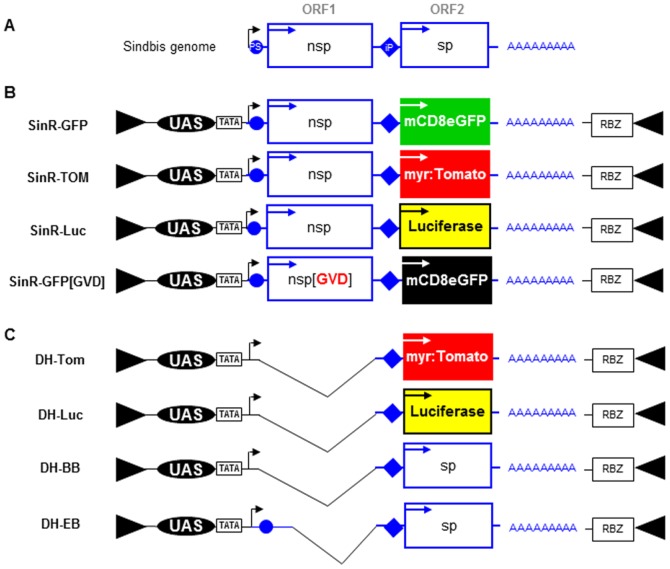
A toolkit of transgenic Sindbis replicons. **A.** Schematic of Sindbis genome, a bicistronic single-stranded RNA with positive polarity: the 5′ end contains a ‘packaging signal’ (PS) for incorporation into the particle. An ‘internal Promoter’ (iP) can be found (on the ‘antigenome’; see Figure S1A) in between the viral ORF's. Abbreviations: nsp  =  ‘non-structural proteins’; sp  =  ‘structural proteins’. **B.** Four transgenic fly strains containing different Sindbis replicons (SinR) stably inserted into the genome. Each transgenic replicon is harboring different reporter genes, or mutations. Abbreviations: UAS  =  GAL4 ‘GAL4 Upstream activating sequence’; TATA  =  hsp70 TATA box; RBZ: Hepatitis Delta Ribozyme; GFP  =  membrane tagged mCD8:eGFP fusion protein; TOM  =  myristoylated Tomato; Luc  =  firefly luciferase; nsp[GVD]  =  point-mutated RNA-dependent RNA Polymerase. **C.** Four replication-incompetent replicons (SinR), all lacking ORF1 due to deletions in the Sindbis genomic DNA sequence, and harboring different sequences in ORF2. Note that DH-EB harbors a smaller deletion, thus retaining a ‘packaging signal’.

The Sindbis genome encodes a positive-stranded 11.7 kb RNA that is both capped and polyadenylated (for review: [14]). Unlike most eukaryotic RNA molecules, the Sindbis genome is bicistronic, containing two open reading frames (ORFs), separated by stop codons (Figure 1A). The 5′ ORF (ORF1) encodes a ‘non-structural’ polyprotein that is translated and cleaved into the four subunits of the Sindbis replicase (nsp1–4), an RNA-dependent RNA polymerase (RdRP). ORF2 encodes a ‘structural’ polyprotein containing the virus glycoproteins, as well as an RNA-binding capsid protein [15]. Sequences at the 5′ end of the genome are important for packaging the genome into the virus particle [16], [17] (‘packaging signal’, PS), while sequences at the 3′ end are important for the initiation of replication by the RdRP [17], [18]. For viral replication to occur, the RdRP first produces a full-length, complementary copy of the genome in (−) orientation (the ‘antigenome’). In order for the second ORF to be translated, an additional, shorter message in (+) orientation (‘subgenomic RNA’) must be generated by the viral replicase, through initiation at an internal “RNA promoter” on the antigenome [19] (for a summary of the Sindbis replication cycle, see Figure S1A). Different gene products and reporter genes have been inserted into ORF2 of Sindbis replicons to study RdRP-dependent viral replication quantitatively, or to express foreign proteins at high levels [9], [20].

The Sindbis replication cycle takes place in the cytoplasm of the host cell, where it is subject to cellular defense pathways, both in vertebrates, as well as in insects [21]. Because of its powerful molecular genetic tools, *Drosophila* represents an attractive model for studying cellular defenses against viruses [22]. Many RNA viruses replicate in *Drosophila*, including Vesicular Stomatitis Virus (VSV) [23], Cricket Paralysis Virus (CPV), Drosophila C Virus (DCV) [24], ‘Yellow Rift Fever Virus’ (YRFV) [25], and Sindbis [7]. Important roles for several cellular pathways in suppressing or promoting viral replication have been identified in *Drosophila*, like the *imd* pathway, [24], the *Jak/Stat* signaling pathway [26], autophagy [23], and RNA interference (RNAi) [27]. While these studies used purified virus particles injected into the hemolymph, transgenic replicons in combination with real-time qPCR was used to quantify the role of the *imd* pathway, antimicrobial peptides, as well as the Akt/Pi3K pathway on replicon expression [10]–[12]. Hence, the transgenic replicon technique serves as a promising alternative for the genetic dissection of factors affecting viral replication *in vivo*, by increasing both reproducibility and tissue-specificity.

Cellular defense pathways are not the only mechanisms restricting viral replication. The competition between closely related virus genomes, known as ‘homologous interference’, or ‘superinfection exclusion’ [28], [29] remains incompletely understood. In cultured cells, Sindbis genomes originating from infectious particles or from injection can exclude each other's replication [28]–[31]. Moreover, even closely related alphaviruses exhibit ‘superinfection exclusion’ [30]. These studies suggested that initial infection with a first virus leads to production of only few (−) orientation ‘antigenomes’ [29], [30]. In addition, cleavage of the replicase polyprotein by a trans-acting protease (nsp2), leads to a loss of replication activity, while transcription of ORF2 from the internal promoter remains unaffected [30], [31]. As a consequence, while ORF1 of the superinfected virus genome is still translated, its RdRP (as well as the pre-existing RdRP from the persistent virus) can no longer produce a ‘subgenomic RNA’ containing the superinfecting ORF2 [30], [32]. However, certain aspects of ‘superinfection exclusion’ remain incompletely understood. For instance, it is unclear whether the exclusion mechanism selects only a single genome or a small number of genomes for replication, and the extent to which host proteins are required for the exclusion process to be effective remains unknown.

We have developed a multi-purpose *Drosophila* toolkit of inducible, transgenic Sindbis replicons for the rapid visualization and quantitative analysis of virus replication *in vivo* with high spatiotemporal precision. We have generated Luciferase expressing replicons that can be used as an alternative to real-time qPCR for the quantification of viral replication in many different tissues, as well as in different mutant backgrounds. We have also generated replication-deficient replicons harboring large deletions spanning virus ORF1, which encodes the viral replicase. With such deficient replicons carrying either a fluorescent protein or Luciferase we can genetically separate both RdRP-production and transcription of ORF2 from ‘subgenomic RNA’, leading to a binary system of trans-complementing replicons. Furthermore, intact, replication-competent transgenic replicons expressing different fluorescent markers (green or red fluorescent proteins) can be used to simultaneously visualize replication of competing replicon populations *in vivo*. Here we describe how replicons that produce either green or red fluorescence stochastically exclude each other's expression when co-expressed under the control of the same cellular promoter. We show that this process is analogous to ‘superinfection exclusion’, making this process amenable to future genetic dissection. We demonstrate the usefulness of this transgenic approach by quantitatively demonstrating that only a single active RdRP molecule per cell must become ‘licensed’ to replicate replicon RNAs, and that this active RdRP has a strong preference for only the message that encoded it. Hence, this toolkit provides an important extension of existing molecular genetic methods for studying different aspects of virus biology in *Drosophila*.

## Materials and Methods

### Fly stocks

The following fly stocks were used: GMR-GAL4 on II (C. Desplan), UAS-mCD8GFP on II and III (L. Luo), UAS-myr:TdTomato on II and III (T. Schwabe), UAS-Luciferase on III (G. Dietzl), *Dcr2[L811fsX]* (R. Carthew), *r2d2* (R. Carthew), *Ago-2[414]* (R. Carthew), NSyb-GAL4 on III (J. Simpson), ElaV-GAL4 (L. Zipursky), repo-GAL4 (M. Silies), r4-GAL4 on III (FlyBase), *Mef2*-GAL4 on III (FlyBase), *btl*-GAL4 on X (M. Krasnow), *cad*-GAL4 on II (FlyBase), *Sgs3*-GAL4 on III (FlyBase), *rh1*-GAL4 on X (Flybase).

### Molecular Biology

Replicon sequences for SinR-GFP and DH-BB were previously published [13]. Standard molecular biology techniques were used to generate the injection constructs for the following new fly stocks: 1) replication-competent Sindbis replicons (SinR-TOM, SinR-Luc), replication-deficient, one point-mutated Sindbis replicon (SinR-GFP[GVD]), and deficient replicons harboring large deletions of ORF1 (DH-TOM, DH-Luc, DH-EB), as well as 2) UAS constructs (UAS-B2, UAS-G(VSV), UAS-ZAP. A more detailed description for of all constructs is available upon request.

#### 1. Cloning of replicon injection constructs

For the generation of transgenic flies expressing replication-competent Sindbis replicons (SinRep) or defective helper constructs harboring large deletions spanning virus ORF1 (DH), the 13x UAS sites from fly injection vector pEP [33] were PCR amplified and fused to a 57 bp oligo containing the 5′ end of the Sindbis genome up to the first Mfe1 restriction site. For replication-competent replicons, the remaining Sindbis sequences including the multiple cloning site and polyA tail from vector SinRep-5 (Toto1101; [4], gift from S. Schlesinger) were then ligated into this site Mfe1/Xho1. For defective helpers harboring large deletions spanning ORF1, sequences from vector DH-BB were used instead ([4], gift from S. Schlesinger). At the 3′ ends of each construct, a 107 bp Hepatitis Delta Virus Ribozyme sequence was introduced for correct termination of the viral RNA, in between the polyA stretch of SinRep5 and the SV40 poly-adenylation region of the fly injection vector pCasper ([35], gift from J. Rose).

For generating the replication-deficient replicon SinR[GVD]-GFP, the predicted polymerase domain containing the GDD amino acid motif [36] of SinR-GFP was modified to GVD, using PCR-based site-directed mutagenesis.
For the generation of replication-competent replicons SinR-TOM and SinR-Luc, the GFP ORF of SinR-GFP was replaced with red fluorescent protein tdTomato (T. Schwabe), or firefly Luciferase (vector GD278, gift from G. Dietzl), using site directed mutagenesis to create the appropriate restriction enzyme sites.
For the generation of deficient replicons DH-Tom and DH-Luc, the virus ORF2 of transgenic DH-BB was first replaced by a multiple cloning site for the introduction of tdTomato or Luciferase. Furthermore, alternative versions of DH-BB were created by using previously published templates from different Sindbis strains: Sindbis genomic clone TE12, (gift of S. Schlesinger) and Sindbis genomic clone MRE16 ([34]; gift of K. Olson), pEGFP-1 (Clontech).

#### 2. Cloning of UAS-constructs

All inserts were ligated into fly injection vector pUAST [37].

UAS-B2: The entire ORF of protein B2 from Flock House Virus was PCR-amplified from a full-length clone (a kind gift from Jamie Williamson, [38]) with the appropriate restriction enzyme sites attached to the PCR primers and ligated into pUAST (Not1/Xba1).
UAS-G(VSV): The entire ORF of the Vesicular Stomatitis Virus (VSV) Glycoprotein was PCR amplified from pBS-G ([35]; a kind gift from John Rose), with the appropriate restriction sites attached to the PCR primers and subcloned using TA cloning (Life Technologies). The product was sequenced and then ligated into pUAST (Not1/Xba1).
UAS-ZAP: The entire ORF of rat antiviral protein ZAP [39] was PCR-amplified from the full length clone NM173045 (ATCC Inc.) with the appropriate restriction sites attached to the PCR primers and subcloned using TA cloning (Life Technologies). The product was sequenced and then and then ligated into pUAST.

### *In vivo* Fluorescence of *Drosophila*

Living flies expressing fluorescent proteins were anesthetized using CO2 and placed under a dissecting scope. Fluorescent images were recorded using an RT slider camera (Diagnostic Instruments, Inc), and images were processed using SPOT software.

### Immunohistochemistry

Brains were fixed for 45 min in 2% paraformaldehyde and blocked in 10% normal goat serum, then incubated with 1∶10 mouse anti-24B10 (Developmental Studies Hybridoma Bank), 1∶2,000 chicken anti-GFP (Abcam), and 10% normal goat serum and detected with goat-anti chicken Alexa 488 (Invitrogen) and goat anti-mouse Alexa 594 (Invitrogen) at a 1∶200 dilution.

Larval eye discs were dissected from wandering 3^rd^ instar larvae and fixed for 20 minutes using 4% Formaldehyde. Primary antibodies used were anti-GFP (Abcam, 1∶2,000), anti-DsRed (Clontech, 1∶1,000) and a polyclonal antibody against Sindbis virus (1∶1,000; gift from Sondra Schlesinger). Secondary antibodies were applied overnight at 4 degrees at a 1∶200 dilution (goat-anti chicken Alexa 488 (Invitrogen), goat anti-mouse Alexa 594 (Invitrogen), and goat anti-rabbit Alexa 594 (Invitrogen)). For the study of replicon co-expression in larval eye discs, GFP- and RFP-positive cells were counted manually and the average number of co-expressing cells was calculated.

### Luciferase assays

For each genotype tested, 3 anesthetized flies were collected in a 1.5 mL Eppendorf tube and frozen at −80 degrees. Flies were then homogenized in 200 µL Reporter Lysis Buffer (Promega), on ice, using a pestle. Fly fly debris was spun down (2 mins, 4000 rpm) at 4 degrees, and the supernatant was transferred to a fresh tube. Each sample was measured 3 times at room temperature (Illumination Laboratory), mixing 6 µL of the homogenate with 50 µL of Luciferase substrate (Promega). 2–4 biological replicates were measured three times and Luciferase counts were averaged.

### Ethics Statement

No human test subjects, or vertebrate animals/cell lines were used in this study.

## Results

### A toolkit of inducible, transgenic Sindbis replicon reporters

Using the inducible, transgenic Sindbis replicons SinR-GFP and DH-BB, we have recently shown that infectious virus particles can be produced *in vivo*, in a cell type-specific manner, in *Drosophila* [13]. Here we describe an extended toolkit of new transgenic Sindbis replicons for the visualization and quantification of viral replication *in vivo*. Sindbis genomic sequences were placed under the control of 14x GAL4 UAS sites [33], together with a Hepatitis Delta virus Ribozyme (RBZ) at the 3′ end to ensure correct termination after the virus polyA tail, when transcribed by the host cell polymerase (see Materials and Methods). These replicons fell into two categories: The first class consisted of four replicons containing the full Sindbis ORF1, as well as 5′ and 3′ sequences necessary for replication and packaging, while different reporter genes were inserted into ORF2 (Figure 1B). With the exception of one point-mutated version (see below), these replicons were therefore capable of self-replication and resembled transgenic GFP replicons, as previously reported [10]–[13]. In order to visualize expression of two competing replicon species independently *in vivo*, we generated a second fluorescent replicon in addition to the existing SinR-GFP. The newly generated SinR-TOM expressed myristoylated Tomato from ORF2, under RdRP control. To allow quantitative measurements of viral replication in different genetic backgrounds we generated a new Luciferase-containing replicon (SinR-Luc). Finally, as a control for viral specificity of replication, we generated SinR-GFP[GVD], a point-mutated form of SinR-GFP, in which a single conserved amino acid change (GDD → GVD) was introduced in the active site of the viral RdRP encoded by ORF1, thereby abolishing its activity [37].

The second class of replicons was always replication-deficient, due to a large deletion spanning most of ORF1 (termed ‘defective helpers’, or DH, following reference [4]) (Figure 1C). These replicons contained either myristoylated Tomato in the place of ORF2 (DH-TOM), or Luciferase (DH-Luc). As a control, we also used the previously published deficient replicon with the original virus ORF2 [13], as well as a closely related construct harboring a smaller deletion that retained the ‘packaging signal’ (DH-EB [4]). In addition to these replicon-based transgenes, we also used three additional UAS constructs for the mis-expression of foreign proteins (Figure S1B). First, we used the previously described UAS-construct for expression of the viral protein B2 from Flock House Virus, a dominant inhibitor of RNAi [13], [38], [40] Second, we generated a UAS-construct for the expression of the glycoprotein from an unrelated RNA virus, Vesicular Stomatitis Virus (VSV), as a control for the mis-expression of viral glycoproteins (UAS-G(VSV). Finally, we generated a UAS-construct for the mis-expression of the mammalian antiviral protein ZAP, which binds directly to the genomic RNAs of Sindbis and other RNA viruses, leading to their degradation in mammalian cells [39], [41].

### Quantification of *in vivo* replicon expression

**Figure 2:**
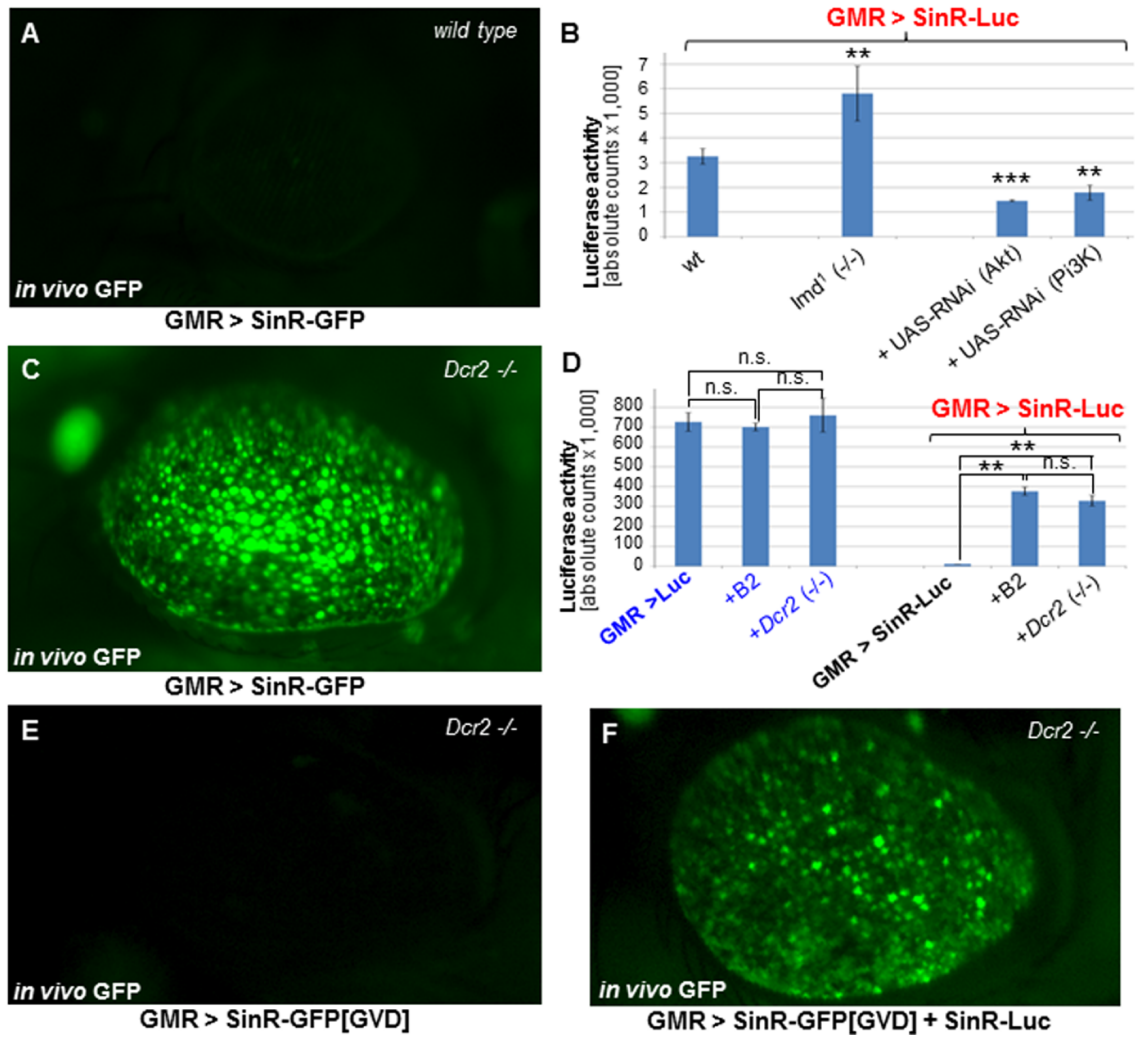
Qualitative and quantitative reporters of *in vivo* viral replication. **A.** Adult, anesthetized flies expressing SinR-GFP using eye-specific driver GMR-GAL4. Virtually no GFP expression is detectable. **B.** Quantification of viral replication *in vivo*, using Luciferase-expressing, replication-competent replicon SinR-Luc. Mutations in *imd* resulted in significantly higher activity. In contrast, knock-down of Akt and Pi3K using UAS-RNAi constructs resulted in a significant decrease. (Luminometer counts in relative units per fly, per µL of homogenate). **C.** Strong GFP expression can be observed in the entire eye, when RNAi is inhibited using homozygous *Dcr2* mutants. **D.** Quantification of RNAi effects on viral replication *in vivo*, using SinR-Luc in combination with different ways of inhibiting RNAi (UAS-B2 co-expression, homozygous *Drc2* mutants). Inhibition of RNAi greatly increased SinR-Luc activity. Note that UAS-Luciferase control levels are unaffected by suppression of RNAi. **E.** SinR-GFP[GVD] with a point mutated RNA-dependent RNA Polymerase never results in GFP expression as detected by *in vivo* fluorescence. **F.** Rescue of GFP expression from SinR-GFP[GVD] in *trans*, using GMR-GAL4, through co-expression of non-fluorescent replicon SinR-Luc, providing an active replicase.

Replication of transgenic replicons expressing fluorescent proteins *in vivo* has previously been reported [10], [13]. We have demonstrated that expression of SinR-GFP in the adult eye is weak, even when using the strong eye-specific driver GMR-GAL4 ([13], [42], Figure 2A), in good agreement with other studies using strong GAL4 drivers in a wild type background [10]. We then used our new Luciferase expressing replicon SinR-Luc to investigate whether Luciferase activity could be used as a reporter of such low viral replication levels *in vivo*. In good agreement with what had previously been reported using a GFP replicon in combination with qPCR [10], we observed a significant increase in Luciferase activity when SinR-Luc was expressed in homozygous mutants lacking *imd*, a crucial component of the fly's innate immunity system (Figure 2B). Furthermore, in agreement with another study [11], we saw a significant reduction in Luciferase activity using GAL4/UAS-mediated, gene-specific RNAi knock-down of the growth regulators Akt or PI3K [43], respectively. Thus, SinR-Luc is a fast and efficient tool for the quantitative investigation of genes affecting viral replication *in vivo*, serving as an attractive alternative to qPCR of viral RNA [10]–[12].

We next applied our new transgenic tools to previous work that demonstrated that the cellular ‘RNA interference’ (RNAi) pathway acts as an important innate immune pathway in the cytoplasm [27]. As previously reported [13], strong fluorescence was obtained using GMR-GAL4 and SinR-GFP when the RNAi pathway was abolished using homozygous *Dcr2* mutants [44], [45] (Figure 2C). Virtually identical results were obtained using other perturbations of the RNAi pathway such as UAS-B2, or mutants in *r2d2* [46], or *Argonaute 2 (Ago2*) [47], [48] (Figure S2A-C). We therefore quantified the effects of RNAi on viral replication using SinR-Luc (Figure 2D). While none of the above perturbations had any significant effect on the expression of a UAS-Luciferase control transgene expressed under the control of GMR-GAL4, we detected a dramatic increase in SinR-Luc activity after co-expression of UAS-B2 (∼38-fold), or in homozygous *Dcr2* mutants (∼33-fold), using GMR-GAL4. Fluorescence was never observed when the point-mutated, replication deficient replicon SinR-GFP[GVD] was over-expressed in homozygous *Dcr2* (-/-) mutants (Figure 2E), a result that was independent of the the method used to inhibit RNAi (Figure S2D,E). However, fluorescence of SinR-GFP[GVD] could be rescued by co-expressing a non-fluorescent, replication-competent replicon (SinR-Luc), thereby providing an active RdRP *in trans* (Figure 2F). Once again, this effect was independent of the means by which RNAi was blocked (Figure S2F, G). Thus, expression from ORF2 of these replicons was driven specifically by the viral RdRP and strongly suppressed by RNAi. In mammals, the zinc-finger antiviral protein ZAP binds directly to the genomic RNAs of Sindbis, leading to its degradation [39], [41]. We therefore tested if ZAP could be used as an additional tool to repress replicon expression in flies, when mis-expressed there. However, over-expression using UAS-ZAP (see materials and methods) had no effect on SinR-GFP expression, either in wild type flies, or in flies in which RNAi was inhibited (Figure S2H–K). Using SinR-Luc in the same backgrounds also revealed no significant difference in replicon expression levels (Figure S2L). Thus, ZAP is likely inactive in flies, most likely due to the absence of cellular factors necessary for ZAP activity. Taken together, these data demonstrate that our transgenic Sindbis replicons provide a powerful toolkit for the qualitative and quantitative analysis of RdRP-driven viral expression *in vivo*.

### Tissue-specific quantification of replicon expression *in vivo*

**Figure 3:**
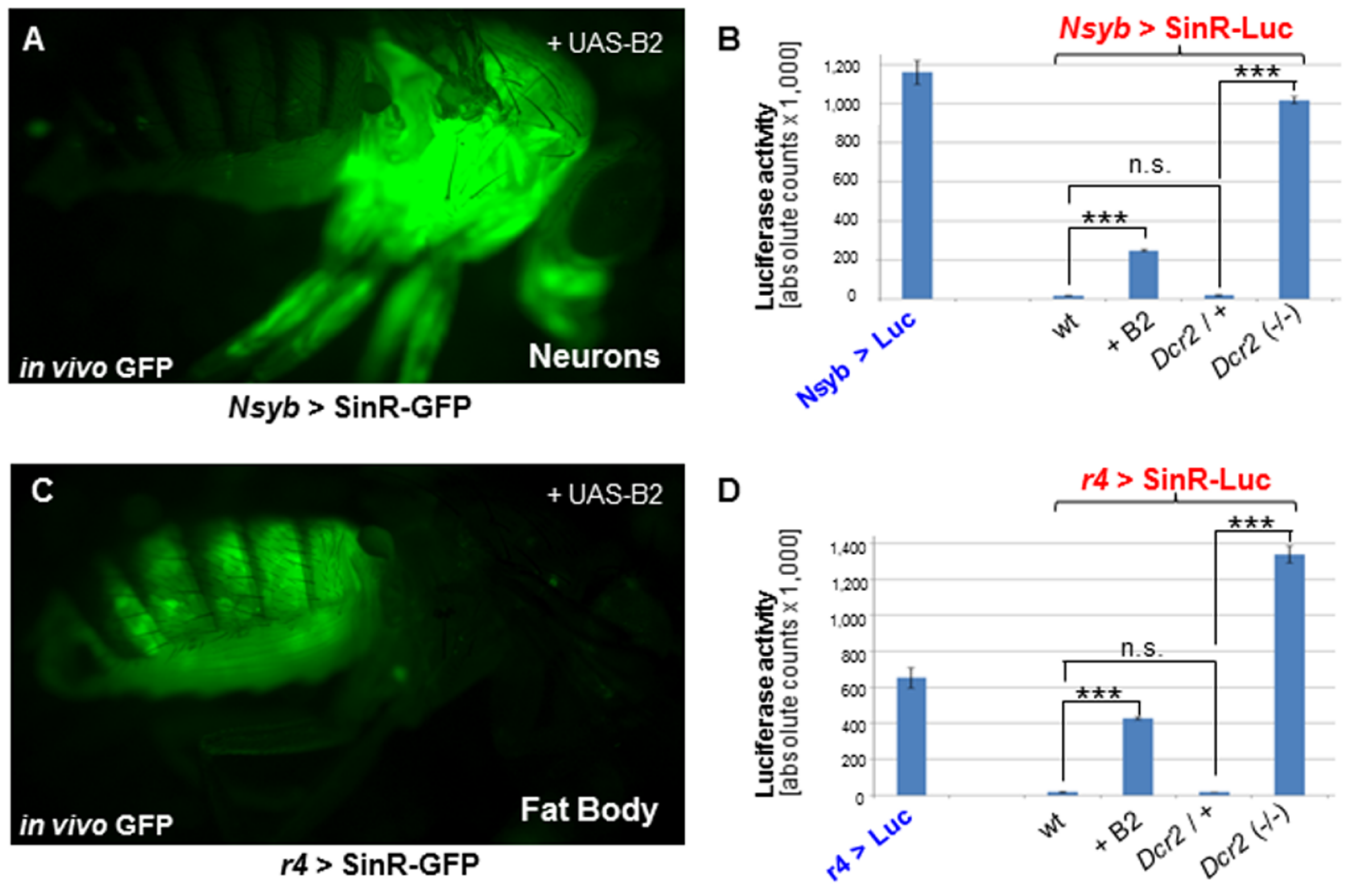
Quantification of Sindbis replicon expression in different tissues. **A,C.** Examples of SinR-GFP expression in different tissues. Labeled are adult neurons (*NSyb*-GAL4; A) or adult fat body (*r4*-GAL4; C). For both tissues, RNAi was inhibited using UAS-B2. **B.** Quantification of SinR-Luc Luciferase activity in neurons. Significantly higher levels of Luciferase activity were obtained in homozygous *Dcr2* mutants, comparable to those obtained with UAS-Luciferase controls. Inactivation of RNAi using UAS-B2 had weaker yet comparable effects while *Drc2* heterozygotes show little to no effect. **D.** Similar effects were obtained in other tissues, like the adult fat body). All luminometer counts in relative units per fly, per uL of homogenate.

An obvious advantage of inducible, transgenic replicons is the possibility of targeting analysis of viral replication to specific tissues. We have previously shown how viral replication can be visualized in different tissues using SinR-GFP in combination with specific GAL4 driver lines ([13] (Figure S3). We now extended these studies by quantifying viral replication in different tissues using SinR-Luc (Figure 3). We chose driver lines expressed in neurons (*NSyb*-GAL4), the adult fat body (*r4*-GAL4), and glial cells (*repo*-GAL4). In all cases, strong expression of GFP was observed only when RNAi was inhibited (Figure 3A, C, E). We quantified virus replication in all three tissues using SinR-Luc (Figure 3B, D; Figure S3G). As before, SinR-Luc activity in all three wild type tissues was very low when compared with UAS-Luciferase controls (1.3% in neurons, 2.6% in fat body, and 18.1% in glia). When we abolished RNAi using homozygous mutants of *Dcr2* (34,35), activity levels became comparable to, or even greater than, UAS-Luciferase controls in all three tissues (87.8% of control levels in neurons, 204% in fat body, and 267% in glia). In all cases, SinR-Luc activity was lower when RNAi was inhibited using UAS-B2, as compared with removal of *Dcr2*, suggesting strong but incomplete inhibition of RNAi using this construct, especially with increasing numbers of UAS constructs in the same fly (21% of UAS-Luciferase levels in neurons; 119% in fat body; 65% in glia). Thus, luciferase replicons serve as an efficient means for the tissue-specific, quantitative study of the effects that cellular defense pathways have on viral replication.

### Trans-activation of defective helper replicons

**Figure 4:**
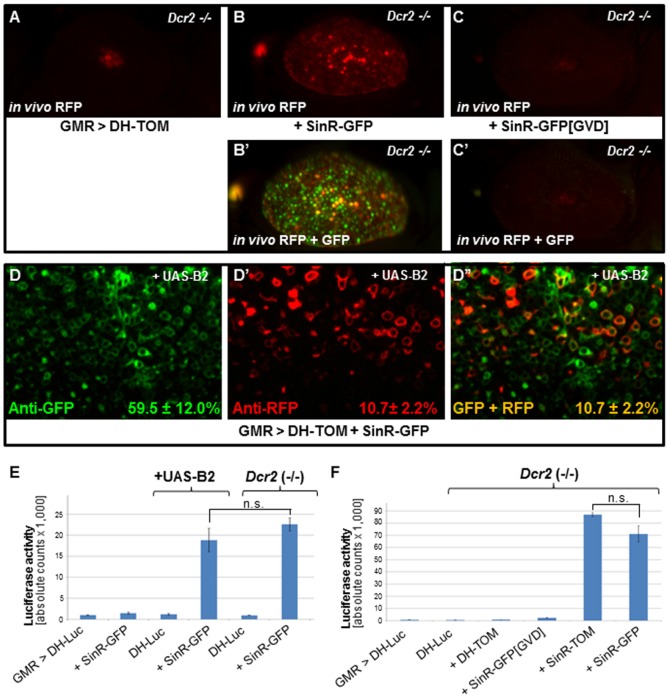
Trans-activation of defective helper replicons. **A.** Expression of replication-defective replicon DH-TOM in the adult eye, using GMR-GAL4 in *Dcr2* homozygotes. Low levels were visible as ‘pseudopupil’, in the center of the eye, most likely due low-level ribosomal read-through (despite numerous nonsense ATG's). **B.** Strong expression of DH-TOM activated in *trans* from a 2^nd^ replicon (SinR-GFP), contributing an intact RdRP. However, red fluorescence is sparse, and co-expression of GFP and Tomato is rare. **C.** The point-mutated replicon SinR-GFP[GVD] always failed to trans-activate defective replicon DH-TOM. **D.** Third instar larval eye discs dissected from flies co-expressing UAS-B2, SinR-GFP, and DH-TOM reveal a low level of myr:Tomato trans-activation (C′). **E.** Luciferase activity (in relative units per fly, per µL of homogenate) of defective DH-Luc in different genetic backgrounds. Significant levels of trans-activation by an intact replicon (SinR-GFP) were observed when RNAi was inactivated (+UAS-B2, or *Dcr2* homozygotes). However, the absolute levels were very low when compared to SinR-Luc activity in the same backgrounds. **F.** Co-expression of replicons with deleted replicase ORF (DH-Tom), or a point-mutation (SinR-GFP[GVD]) never trans-activated Luciferase activity of DH-Luc.

We next sought to visualize and quantify the activity of the RdRP acting *in trans* on the internal promoter of the ‘subgenomic RNA’ *in vivo*, by analogy to previous cell culture studies [49]. As expected, expression of the replication-deficient replicon DH-TOM in the adult eye in *Dcr2* (-/-) mutants using GMR-GAL4 resulted in a low level of background expression (Figure 4A), likely due to read-through of the mRNA by ribosomes. However, when an intact, green-fluorescing replicon (SinR-GFP) was co-expressed with DH-TOM in *Dcr2* homozygotes, robust red (and green) fluorescence was observed in the adult eye (Figure 4B). Thus, the RdRP derived from ORF1 of the “green” replicon can drive replication of the deficient “red” replicon. Red fluorescence in absence of GFP was never observed. However, trans-activation of DH-Tom was incomplete, as many ommatidia expressed GFP but not Tomato. Virtually identical results were obtained after inhibiting RNAi in various ways (Figure S4B, E). As expected, the point-mutated replicon SinR-GFP[GVD] never trans-activated the red fluorescence encoded by DH-TOM (Figure 4C; Figure S4C, F). Since the fluorescence signal visualized in any given ommatidium represents the combined expression from eight neuronal photoreceptors as well as non-neuronal support cells, we decided to investigate GFP/Tomato co-expression at higher resolution. To extend these studies to the single cell level, we stained third instar larval eye discs expressing DH-Tom and SinR-GFP (+UAS-B2) under GMR-GAL4 control, using immunohistochemistry and confocal microscopy (Figure 4D). In agreement with the observations of fluorescent ommatidia, only 10.7%+/−2.1% of SinR-GFP expressing cells stained with Anti-GFP co-expressed DH-TOM. We then used DH-Luc to quantify the efficiency of this low-level trans-activation (Figure 4E, F). Since this replicon was replication-deficient due to a large deletion spanning ORF1, detectable levels of Luciferase activity were measured only when RNAi was inhibited and an intact replicon was co-expressed (DH-Luc + SinR-GFP), thereby providing an active RdRP in *trans*. However, the observed levels of expression were only 14.8% of those measured with SinR-Luc activity (where the RdRP acts *in cis*, see Figure S4G for a direct comparison in the same genetic background). Consistent with our expectations, the point-mutated SinR-GFP[GVD], or defective replicons (DH-TOM), could not trans-activate DH-Luc (Figure 4F). Thus, trans-activation of defective helpers occurred, but was inefficient, even as activation of ORF2 *in cis* to the active RdRP remained efficient.

### Stochastic exclusion between launched transgenic replicons

**Figure 5:**
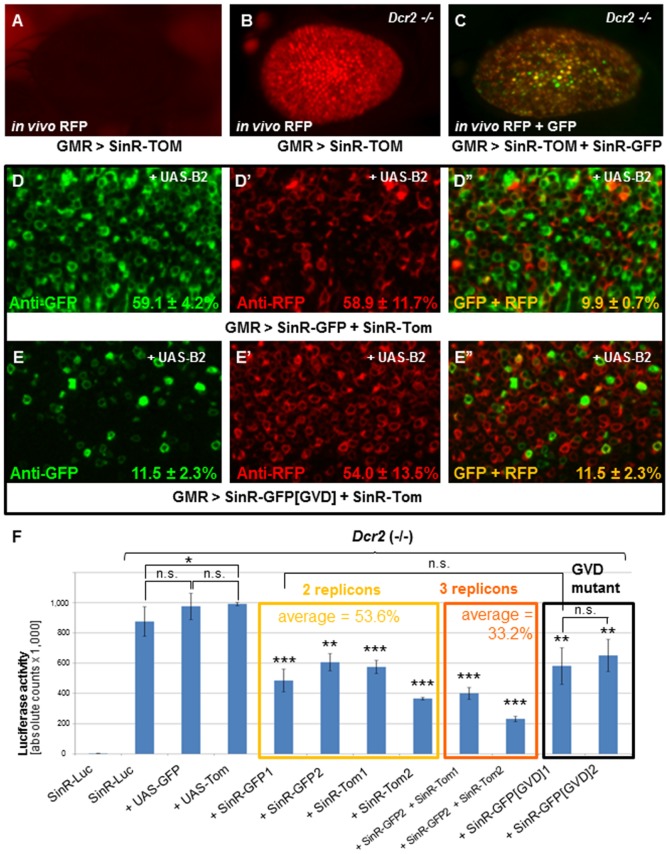
Stochastic exclusion between transgenic replicons. **A.** Expression of red fluorescent, replication-competent replicon SinR-Tom was undetectable when driven in the adult eye using GMR-GAL4, with the RNAi pathway intact. **B.** Strong levels of Tomato expression from SinR-TOM observed in homozygous *Dcr2* mutants. **C.** Strong red and green fluorescence in flies co-expressing both SinR-GFP and SinR-TOM in *Drc2* mutants. Note that not all ommatidia co-expressed the two replicons. **D.** Third instar larval eye discs dissected from flies co-expressing SinR-GFP, SinR-TOM, and UAS-B2 under GMR-GAL4 control. Strong co-expression was observed only in ∼10% of ommatidia. Apparently, the two replicons exclude each other's expression. **E.** Third instar larval eye discs co-expressing replication-deficient SinR-GFP[GVD] and SinR-TOM revealing a similarly low degree of GFP trans-activation. In these cases, the two proteins always co-localized, as expected. **F.** Quantification of *in vivo* replicon exclusion using SinR-Luc. In homozygous *Dcr2* mutants, SinR-Luc activity was not affected by co-expression of either UAS-mCD8GFP, or UAS-myr:Tomato. However, co-expression of different insertions of SinR-GFP (suffix 1 and 2) or SinR-Tom all reduced Luciferase levels by approximately half (−46.7%). Moreover, expression of all three replicons in *Dcr2* mutants lowered the Luciferase counts by approximately two thirds (−66.8%). Interestingly, co-expression of replication-deficient SinR[GVD]-GFP, also reduced Luciferase activity (−30%).

Cultured cells infected with Sindbis cannot be re-infected with a second related virus, a phenomenon known as ‘superinfection exclusion’, or ‘homologous interference’ [28], [29]. We therefore tested if exclusion between replicons was the reason why trans-activation was inefficient. As with SinR-GFP, fluorescence of replication-competent SinR-TOM in the adult eye was strongly inhibited by the RNAi pathway (Figure 5A, B, Figure S5A–C). When both SinR-GFP and SinR-TOM were co-expressed, many ommatidia were labeled with only one of the two colors (Figure 5C), independent of the genetic background inhibiting RNAi (Figure S5D–F). To investigate co-expression of SinR-GFP and SinR-TOM at a single-cell level, we dissected larval eye discs labeled with antibodies against GFP and RFP/DsRed (Figure 5D). Remarkably, only 9.9%+/−0.7% of the labeled cells expressed high levels of both fluorescent proteins. For comparison, we also co-stained larval eye discs dissected from flies co-expressing the point-mutated SinR-GFP[GVD] and SinR-TOM under GMR-GAL4 control (Figure 5E). In this genetic combination, green fluorescence could only be obtained through trans-complementation via SinR-TOM providing the active RdRP. Indeed, only very few GFP-positive cells were observed, and these always co-localized with the red fluorescent epitope (as 11.5%+/−2.3% of the SinR-TOM-positive cells expressed GFP, and thus exhibited transactivation). Thus, co-expression of two replication-competent replicons within the same cell occurred at the same low rate than trans-activation of a replication-deficient replicon. Finally, as a control, we stained eye imaginal discs co-expressing UAS-mCD8:GFP and UAS-myr:Tomato (Figure S5G). As expected, the fluorescent proteins always co-localized in every cell.

Using SinR-Luc, we quantified this replicon exclusion (Figure 5F). When SinR-Luc was co-expressed with either SinR-GFP or SinR-TOM, Luciferase activity was reduced by almost exactly half, on average (53.6%), consistent with half the cells expressing SinR-Luc (independent of the method used to inhibit RNAi; Figure S5H). Furthermore, co-expression of all three replicons (SinR-Luc + SinR-GFP + SinR-TOM) resulted in almost exactly one third of SinR-Luc activity (33.2%), consistent with each cell stochastically choosing to express only one of the three replicons. As controls, co-expression of SinR-Luc with UAS-mCD8:GFP or UAS-myr:Tomato had no effect on Luciferase activity levels. We also confirmed that co-expression of a replicon did not affect Luciferase activity in general, since activity of UAS-Luciferase was not affected (Figure S5I). Finally, the point-mutated SinR-GFP[GVD] also reduced the activity of SinR-Luc significantly, suggesting that a replication competent RdRP is not required for a replicon's ability to exclude another replicon. Taken together, we conclude that different transgenic replicons stochastically avoid co-expression within the same cell, demonstrating behavior analogous to ‘superinfection exclusion’ previously described for virus particles in cell culture [28]–[30], [32].

### The structural ORF2 has no influence on exclusion

**Figure 6:**
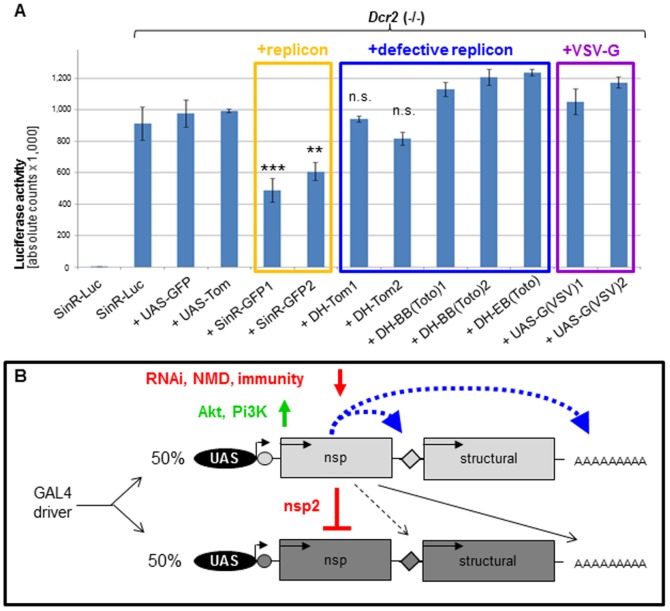
ORF2 does not induce exclusion and Model. **A.** Quantitative test whether defective helper transgenes excluded replicon expression, using SinR-Luc. In homozygous *Dcr2* mutants, expression levels of SinR-Luc were unaffected by co-expression of either DH-TOM, DH-BB or DH-EB defective replicons, all harboring deletions of ORF1. Co-expression of foreign glycoproteins (UAS-G[VSV]) also had no significant effect on Luciferase expression. **B.** Model summarizing factors regulating *in vivo* replicon expression. While replicon expression is inhibited by cellular pathways (RNAi, NMD, innate immunity), a strong preference of the viral RdRP for the internal promoter on the ‘subgenomic RNA’ originating from the same transcript exists. As a result, trans-activation is weak, even from transgenes with a deleted RdRP.

Since the replication-incompetent, point-mutated replicon SinR-GFP[GVD] also excluded SinR-Luc, we tested whether expression from ORF2 in absence of an ORF1 was sufficient for the exclusion process. If so, then defective helpers with large deletions spanning ORF1 should also repress SinR-Luc activity. However, two different genomic inserts of DH-TOM had no effect on Luciferase levels from SinR-Luc, when co-expressed using GMR-GAL4 (Figure 6A). We also tested whether a potential role of ORF2 in ‘superinfection exclusion’ was dependent on encoding the viral ‘structural proteins’, instead of a foreign reporter gene. However, co-expression of SinR-Luc with transgenic ‘defective helper’ replicons DH-BB or DH-EB [4], [13], which harbor different sized deletions in ORF1 (see Materials and Methods), also had no effect on Luciferase activity (Figure 6A). Consistent with these results, co-expression of a foreign glycoprotein from ‘Vesicular Stomatitis Virus’ (VSV) also had no effect on Luciferase activity. Thus, in these constructs, the sequences responsible for replicon exclusion are deleted, and the capacity to exclude another replicon is lost together with ORF1.

## Discussion

### A transgenic replicon toolkit for *Drosophila*

We have developed a versatile toolkit of transgenic replicons that enable different aspects of Sindbis virus biology to be studied *in vivo*. We have previously shown that the replication-competent replicon SinR-GFP can be used in combination with DH-BB, to produce infectious viral particles *in vivo*, through self-assemby in *trans*, entirely from transgenes [13]. This process remains inefficient, most likely due to the low efficiency of DH-BB trans-complementation *in vivo* (discussed in detail below; Figure S6A). The use of alternative helper transgenes (see Materials and Methods), as well as future genetic screens, will enable systematic improvement of this technique. We have shown that SinR-GFP and SinR-TOM allow visualizing the expression levels of these two replicon populations when co-expressed, thereby rendering competition between viral genomes amenable to direct genetic dissection. Using the point-mutated GFP replicon SinR-GFP[GVD], or the ‘deficient’ Tomato replicon DH-TOM harboring a large deletion making it replication-incompetent, we have visualized replicase activity *in trans*, thereby providing a binary system for studying viral transcription from the ‘subgenomic RNA’ *in vivo*. These transgenic GFP/TOM replicons provide a fast and powerful *in vivo* system for large-scale mutagenesis screens searching for host factors affecting different aspects of virus replication.

The luciferase replicons we have developed (SinR-Luc, DH-Luc) provide an attractive alternative for high-throughput quantification of many genetic phenotypes. Luciferase replicons have been developed for quantifying the replication of medically relevant RNA viruses like West Nile [50] or Dengue [51]. However, none of these replicons have been used as transgenic, inducible versions in their insect host. Using SinR-Luc, we have quantified the effects of cellular defense pathways on viral replication, using different mutations in the RNAi pathway. We also showed that knock-down of specific host genes can produce negative (*imd*), as well as positive (*Akt*) effects on viral replication, in agreement with previous studies [10]–[12]. Furthermore, since there are genome-wide UAS-RNAi collections available [43], the SinR-Luc replicons we present here provide a straightforward tool for systematic, unbiased genetic screens for factors affecting viral replication *in vivo* in the entire animal, as well as in specific tissues. This approach may serve as an attractive alternative to other genome-wide approaches using injected particles [25] or microarray data [12]. This potential extends to the deficient Luciferase replicon DH-Luc, allowing the identification of host factors that affect trans-complementation. Alternatively, DH-Luc could also be used to quantify trans-activation by RdRP molecules provided by purified virus particles, which can be introduced into flies either orally or via injection. Hence, based on what we have shown here, the future use of transgenic Luciferase expressing replicons derived from other, more medically relevant arboviruses will be very promising. Of particular importance is the possibility to produce transgenic Mosquito strains that could carry such transgenic tools [52], and might enable even more powerful *in vivo* screens for controlling the diseases caused by these viruses.

### Stochastic exclusion between replicons *in vivo*

We have used red- and green-fluorescent replicons to visualize stochastic exclusion between these replicons, analogous to ‘superinfection exclusion’, or ‘homologous interference’, previously observed in cultured cells [28]–[30], [32]. This widespread phenomenon, which remains incompletely understood, has been described for many different RNA viruses, including Dengue [53], Hepatitis C Virus [54], Rubella virus [55], and West Nile [56]. We co-expressed red and green-fluorescent replicons *in vivo*, under the control of cellular RNA polymerase II from the same promoter. However, in spite of this strong expression, our data demonstrate that only one of these two messages is strongly replicated by RdRP, in any given cell. As a result, the exclusion process must stochastically select a single viral RNA per cell, resulting in a ‘salt and pepper’ pattern across a population of cells. We show that a replicon lacking ORF1 cannot be selected in this stochastic process, and that individual replicase molecules prefer the RNA that encoded them. Only on rare occasions does the RdRP accept the ‘foreign’ replicon RNA as a template, resulting in low levels of co-expression (∼10%). As a consequence, the number of cells expressing defective helper transgenes, which crucially depend on trans-activation, was low.

Combining our data with the previous studies of superinfection exclusion [30] (Figure S6B) reveals important new aspects of this model (Figure 6B). First, the presence of two RdRP-bearing viral replicons appears to lead to a competition in which only one RdRP molecule becomes stochastically “licensed” to be active. As previously suggested [30], we propose that the first polyprotein molecule to be translated immediately begins the exclusion process by proteolytic inactivation of all other replicase molecules in the cell. Second, this active RdRP strongly prefers to transcribe the (−) antigenome to the RNA that it originated from, presumably through close physical contact in a cytoplasmatic protein/RNA complex [57]. Using point-mutated replicons and deficient helpers, we show that at the same low frequency (∼10%), either a second RdRP gets licensed, or a single RdRP can replicate a second viral mRNA. As a result of these two mechanisms, we conclude that only one RNA molecule per cell is typically selected for replication. We note that this model is also entirely consistent with our observations, and those of others, that point-mutated replicons (but not ones harboring deletions of ORF1) can mediate exclusion, as SinR-GFP[GVD] still produces an important cleavage product, the protease nsp2. Moreover, these studies provide genetic support for the observed isolation of replication complexes within membrane bound intracellular organelles, as it is possible that the single, licensed genome (and its descendants) could occupy such vesicular structures [57]. While we favor this model, we cannot exclude specific variations of alternative models that have been proposed for other viruses, including a possible competition for host factors, or the involvement of virus proteins [58].

### Concluding Remarks

Taken together, the collection of transgenic replicons we present here serves as a useful toolkit for the *in vivo* study of RNA virus replication in a genetically tractable insect host. This toolkit adds important new aspects, as well as powerful alternative strategies, to what is currently available as resources for the visualization and quantification of viral replication in general, as well as very specific aspects of the regulation of replication, including phenomena like superinfection exclusion. Thus, in combination with our recent progress towards producing infectious particles *in vivo* through particle launching from inducible transgenes [13], the toolkit presented here now makes many different aspects of viral replication accessible to genetic analysis. Moreover, we believe that this approach can be generalized to establish transgenic animal models of other highly medically relevant RNA viruses, most of which are insect-borne, and against which efficient counter-measures are still missing. In the future, such efforts will not be limited to transgenic *Drosophila*, since Mosquitoes, the natural vectors for many of these viral pathogens, have now become amenable to molecular genetic manipulation as well, promising the development of even more powerful transgenic tools [52], [59]. Thus, such studies based on transgenic replicons have the potential for leading towards important progress in basic biology, as well as pharmaceutical applications.

## Supporting Information

Figure S1 **The Sindbis replicon cycle and trans-activation.** **A.** Schematic representation of the bi-cistronic wild type Sindbis genome (in blue), and summary of the virus replication cycle. Note that expression of Sindbis ORF2 depends on at least one round of replication of the genome, since production of its message, the ‘subgenomic RNA’, depends on the presence of the ‘antigenome’, i.e. the complementary strand copy of the Sindbis genome. Abbreviations: UAS  =  GAL4 ‘upstream activating sequences’, RdRP  =  RNA-dependent RNA Polymerase, blue circles, ‘PS’: packaging signal for the incorporation of the Replicon RNA into the virus particle, blue square, ‘iP’: internal RNA-dependent promoter recognized by the viral RdRP. **B.** Three UAS-contructs generated for this study: UAS-G(VSV) expressed the Glycoprotein from Vesicular Stomatitis Virus (VSV) under UAS control. UAS-B2 was generated for the dominant, cell-type specific suppression of RNAi, using viral protein B2 from Flock House Virus [13]. UAS-ZAP expresses antiviral protein ZAP cloned from rats.
(TIF)

Figure S2 **Additional characterization of GFP replicons.** **A–C.** Viral RdRP-driven expression of mCD8:eGFP from SinR-GFP transgenes, expressed in the adult eye using GMR-GAL4. Shown are three additional ways of inhibiting RNAi (from left to right): homozygous *Ago2* mutants (A), dominant suppression of RNAi using UAS-B2 transgenes (B), and homozygous *r2d2* mutants (C) (see materials and methods). **D–G.** Additional characterization of point-mutated SinR-GFP[GVD]: When expressed with GMR-GAL4, no mCD8:eGFP expression was observed UAS-B2 was over-expressed (D), or in homozygous *r2d2* mutants (E). Expression of mCD8:eGFP could be rescued by co-expression of non-fluorescent, replication-competent SinR-Luc, providing a wild type copy of RdRP in *trans*, both when UAS-B2 was used to suppress RNAi (F), or in *r2d2* mutants (G). **H–L.** Testing the antiviral potential of the zinc finger antiviral protein ZAP in *Drosophila*. In mammals, ZAP was shown to directly bind to Sindbis genomic RNA, leading to its degradation (H). pUAST-ZAP transgenes generated for producing transgenic UAS-ZAP flies (see materials and methods) (I). Over-expression of the ZAP in all photoreceptors, using GMR-GAL4, UAS-B2, and UAS-ZAP transgenes, had no effect on mCD8:eGFP expression *in vivo* (J,K). Over-expression of two different insertions of UAS-ZAP transgenes also had no significantly inhibiting effect on viral transcription as measured using SinR-Luc (L).
(TIF)

Figure S3 **Replicon expression in diverse tissues.** **A.–E.** Examples of viral expression *in vivo*, in different tissues. Labelled tissues are adult gut (*cad*-GAL4; A) muscles (*mef2*-GAL4; B), larval salivary glands (*Sgs3*-GAL4; C), adult photoreceptors (*rh1*-GAL4; D), and pupal trachea (*btl*-GAL4; E). For each tissue, expression of SinRep-mCD8eGFP in combination with UAS-B2 is shown. Viral expression largely recapitulated expression of the marker gene, as visualized with UAS-eGFP (not shown). **F, G.** Additional quantification of replicon expression in glia: replicon expression is driven by *repo*-GAL4 (SinR-GFP; F). Luminometer counts (in relative units per fly, per uL of homogenate) of *repo*-GAL4 driving SinR-Luc (G) in different genetic backgrounds inhibiting RNAi (same as in Figure 3B, D).
(TIF)

Figure S4 **Trans-activation of defective reporter replicons.** **A.** Expression of the DH-TOM defective replicon in the adult eye, when driven with GMR-GAL4 in homozygous *r2d2* mutants. Weak expression is seen in the ‘deep pseudopupil’. **B.** Strong levels of myr:Tomato expression from the defective replicon activated in *trans*, from a 2^nd^ GFP-expressing replicon (SinR-GFP) contributing a wild type RdRP in *trans*, in r2d2 mutants. C. Under the same conditions, the point-mutated, replication-deficient replicon SinR-GFP[GVD] fails to activate Tomato expression in *trans*. **D–F.** Same experiments as above, using UAS-B2 over-expression to inactivate the RNAi pathway. **G.** Direct comparison of Luciferase activity levels (Luminometer counts per µL homogenate, per fly), of GMR-GAL4 driving expression of UAS-Luc, SinR-Luc, and DH-Luc in wild type flies, in *Dcr2* homozygotes, as well as when the GFP replicon SinR-GFP is co-expressed in *Dcr2* mutants (red boxes). All numbers were re-plotted from previous graphs, for better comparison. Note that trans-activation of DH-Luc results in very low activity levels.
(TIF)

Figure S5 **Superinfection exclusion of Sindbis particles.** **A–C.** Additional genotypes to block the RNAi pathway and enable strong levels of SinR-TOM expression in the adult eye, using GMR-GAL4 (from left: *r2d2* homozygotes (A), *Ago2* homozygotes (B), and UAS-B2 co-expression (C). **D–F.** Same genotypes co-expressing two replication-competent replicons, SinR-GFP and SinR-TOM, using GMR-GAL4. Many ommatidia choose expression of one replicon over the other (+UAS-B2 eyes were rough and therefore more difficult to analyze). **G.** Third instar larval eye discs dissected from control flies co-expressing UAS-mCD8GFP and UAS-myr:tdTomato reporter constructs, as well as UAS-B2 under the control of GMR-GAL4. Widespread co-localization of the two fluorescent proteins was observed. **H.** Exclusion between replicons is independent of the means by which the RNAi pathway is inactivated: the same reduction in Luciferase activity induced by SinR-GFP was observed when co-expressing UAS-B2, in *Dcr2* homozygotes, and in *r2d2* homozygotes. Activity levels in comparison to UAS-Luciferase are shown for comparison (same data as Figure S1). **I.** Activity of UAS-Luciferase was not affected by co-expression of Sindbis replicons. Neither co-expression of UAS-B2, nor homozygous (or heterozygous) mutations in *Dcr2* affected expression levels, when driven with GMR-GAL4.
(TIF)

Figure S6 **Trans-activation of Sindbis structural proteins and Model.** **A.** Third instar larval eye discs dissected from flies co-expressing SinR-GFP and defective helper DH-BB under the control of GMR-GAL4. Staining with an Antibody against Sindbis (see materials and methods) revealed sparse expression in developing neurons posterior to the morphogenetic furrow (red), where the driver is expressed. **B.** Summary of RdRP polyprotein cleavage by the nsp2 protease. Note that excess protease activity will abolish RdRP's replication activity, while transcription from the internal promoter on existing antigenomes remains active [30]. **C.** Additional Model Figure supporting Main Figure 6B, displaying the inability of deficient replicons harboring large deletions spanning ORF1, to induce stochastic exclusion.
(TIF)
