# Supplementary figures and images for: A Drosophila Toolkit for the Visualization and Quantification of Viral Replication Launched from Transgenic Genomes
Source: PLoS One. 2014 Nov 11;9(11):e112092. doi: 10.1371/journal.pone.0112092 (PMC4227818; doi:10.1371/journal.pone.0112092)

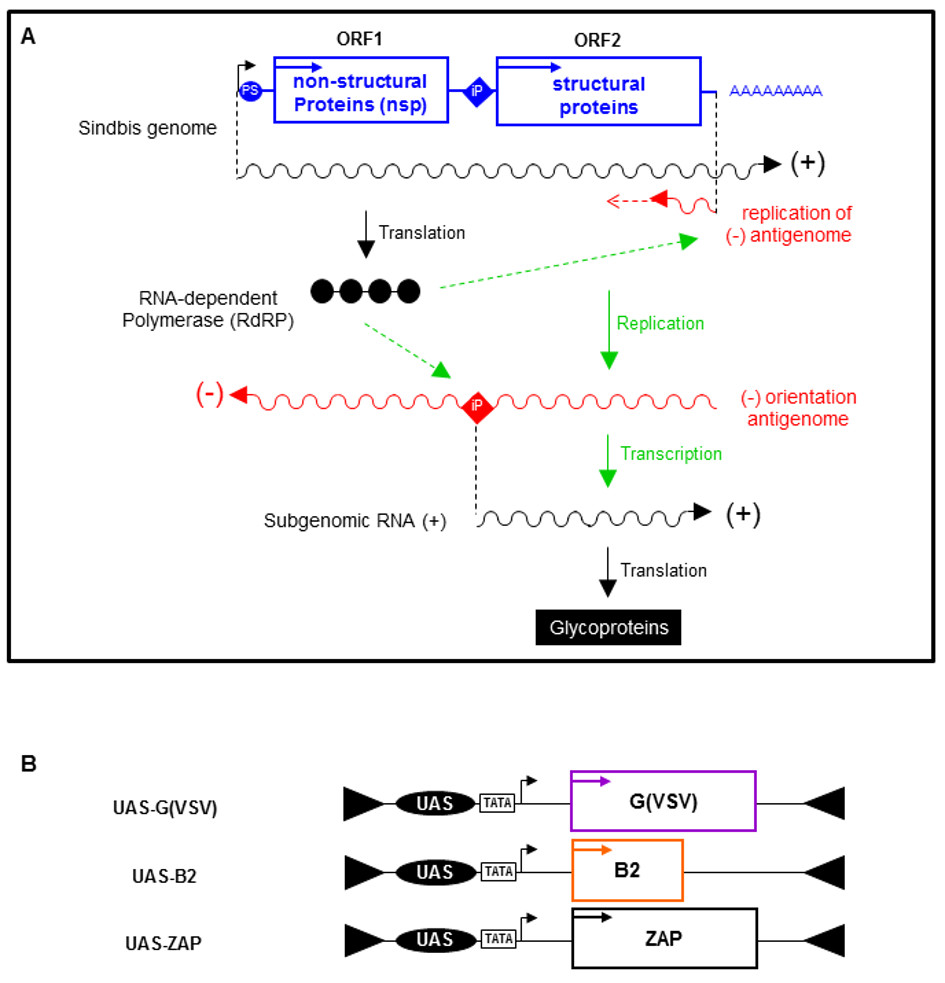

Supplement: Figure S1 — The Sindbis replicon cycle and trans-activation. A. Schematic representation of the bi-cistronic wild type Sindbis genome (in blue), and summary of the virus replication cycle. Note that expression of Sindbis ORF2 depends on at least one round of replication of the genome, since production of its message, the ‘subgenomic RNA’, depends on the presence of the ‘antigenome’, i.e. the complementary strand copy of the Sindbis genome. Abbreviations: UAS = GAL4 ‘upstream activating sequences’, RdRP = RNA-dependent RNA Polymerase, blue circles, ‘PS’: packaging signal for the incorporation of the Replicon RNA into the virus particle, blue square, ‘iP’: internal RNA-dependent promoter recognized by the viral RdRP. B. Three UAS-contructs generated for this study: UAS-G(VSV) expressed the Glycoprotein from Vesicular Stomatitis Virus (VSV) under UAS control. UAS-B2 was generated for the dominant, cell-type specific suppression of RNAi, using viral protein B2 from Flock House Virus [13]. UAS-ZAP expresses antiviral protein ZAP cloned from rats. (TIF) [file pone.0112092.s001.tif]

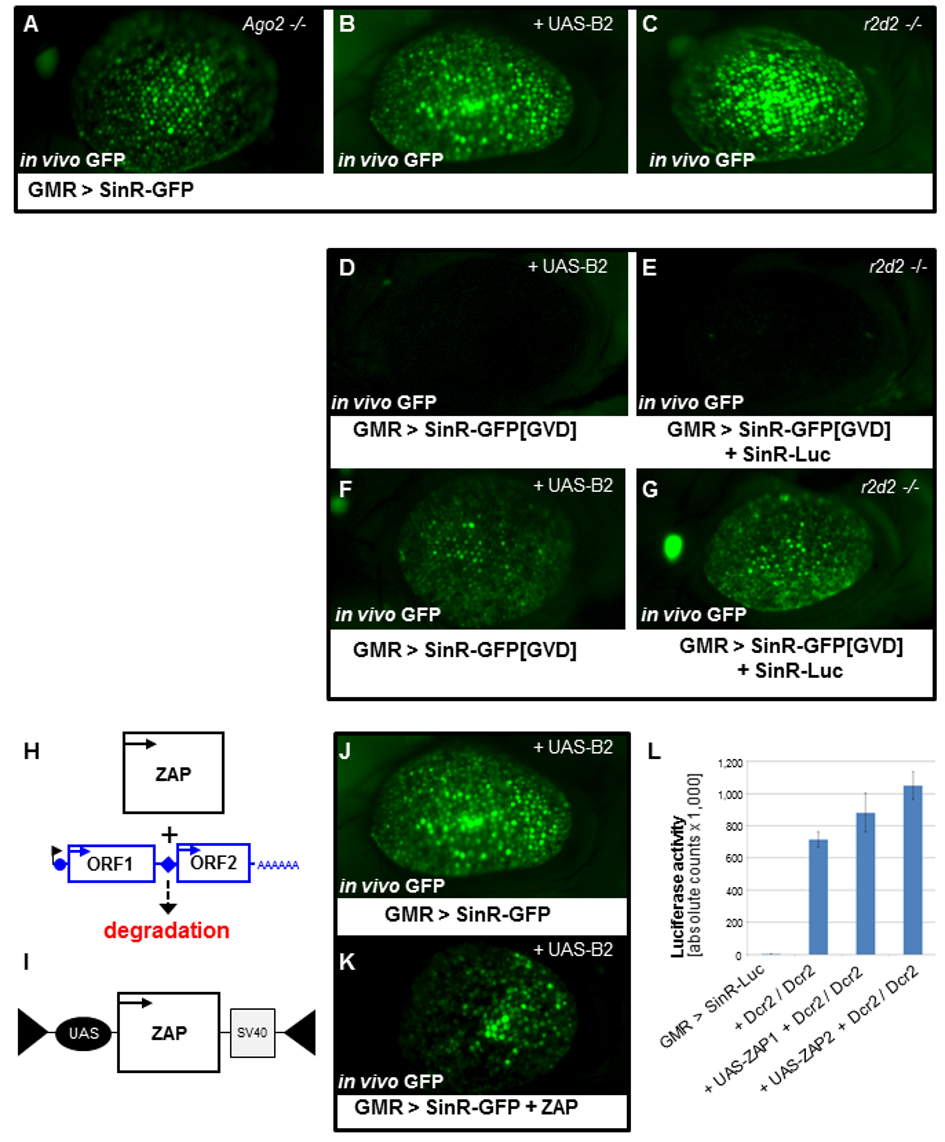

Supplement: Figure S2 — Additional characterization of GFP replicons. A–C. Viral RdRP-driven expression of mCD8:eGFP from SinR-GFP transgenes, expressed in the adult eye using GMR-GAL4. Shown are three additional ways of inhibiting RNAi (from left to right): homozygous Ago2 mutants (A), dominant suppression of RNAi using UAS-B2 transgenes (B), and homozygous r2d2 mutants (C) (see materials and methods). D–G. Additional characterization of point-mutated SinR-GFP[GVD]: When expressed with GMR-GAL4, no mCD8:eGFP expression was observed UAS-B2 was over-expressed (D), or in homozygous r2d2 mutants (E). Expression of mCD8:eGFP could be rescued by co-expression of non-fluorescent, replication-competent SinR-Luc, providing a wild type copy of RdRP in trans, both when UAS-B2 was used to suppress RNAi (F), or in r2d2 mutants (G). H–L. Testing the antiviral potential of the zinc finger antiviral protein ZAP in Drosophila. In mammals, ZAP was shown to directly bind to Sindbis genomic RNA, leading to its degradation (H). pUAST-ZAP transgenes generated for producing transgenic UAS-ZAP flies (see materials and methods) (I). Over-expression of the ZAP in all photoreceptors, using GMR-GAL4, UAS-B2, and UAS-ZAP transgenes, had no effect on mCD8:eGFP expression in vivo (J,K). Over-expression of two different insertions of UAS-ZAP transgenes also had no significantly inhibiting effect on viral transcription as measured using SinR-Luc (L). (TIF) [file pone.0112092.s002.tif]

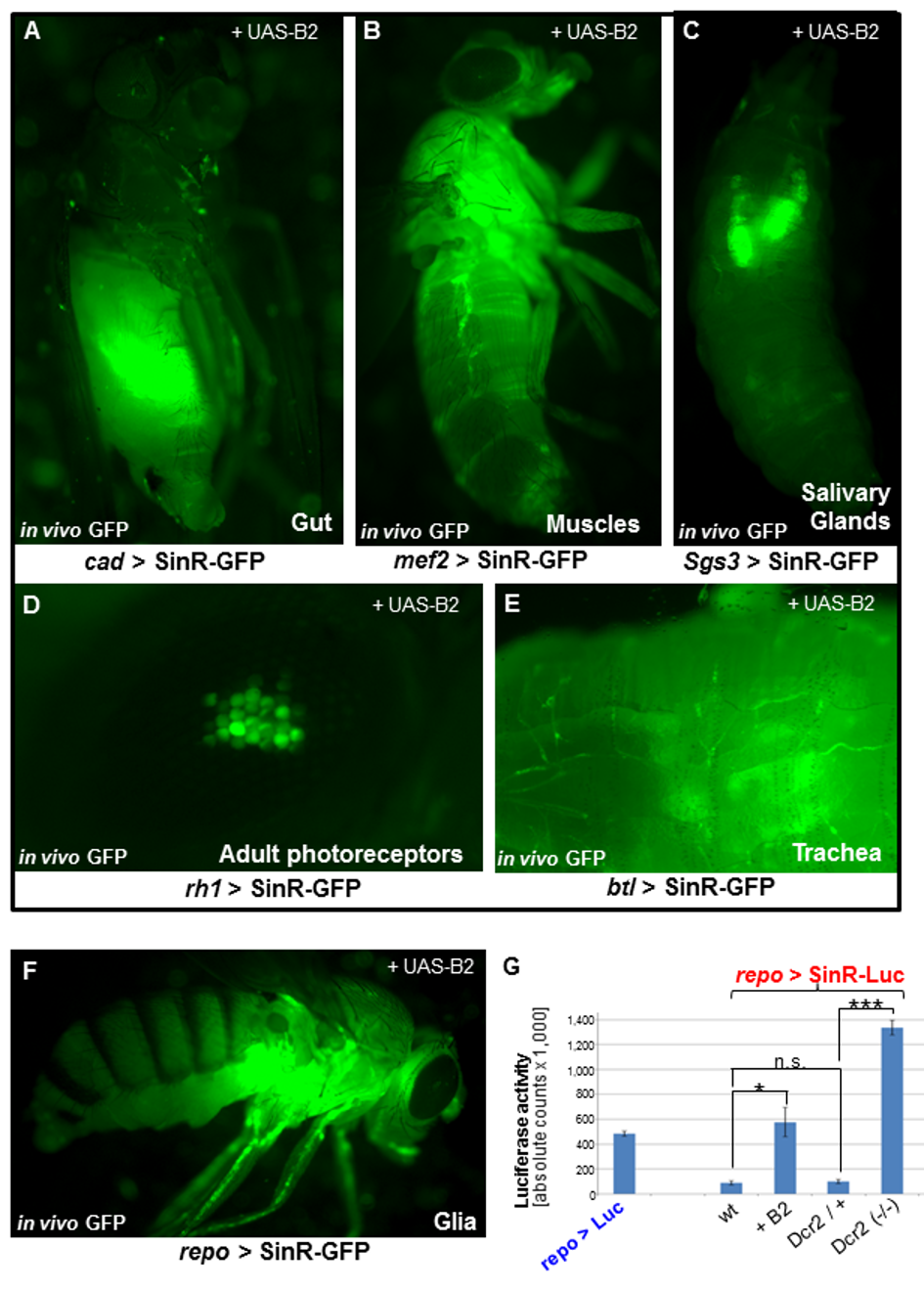

Supplement: Figure S3 — Replicon expression in diverse tissues. A.–E. Examples of viral expression in vivo, in different tissues. Labelled tissues are adult gut (cad-GAL4; A) muscles (mef2-GAL4; B), larval salivary glands (Sgs3-GAL4; C), adult photoreceptors (rh1-GAL4; D), and pupal trachea (btl-GAL4; E). For each tissue, expression of SinRep-mCD8eGFP in combination with UAS-B2 is shown. Viral expression largely recapitulated expression of the marker gene, as visualized with UAS-eGFP (not shown). F, G. Additional quantification of replicon expression in glia: replicon expression is driven by repo-GAL4 (SinR-GFP; F). Luminometer counts (in relative units per fly, per uL of homogenate) of repo-GAL4 driving SinR-Luc (G) in different genetic backgrounds inhibiting RNAi (same as in Figure 3B, D). (TIF) [file pone.0112092.s003.tif]

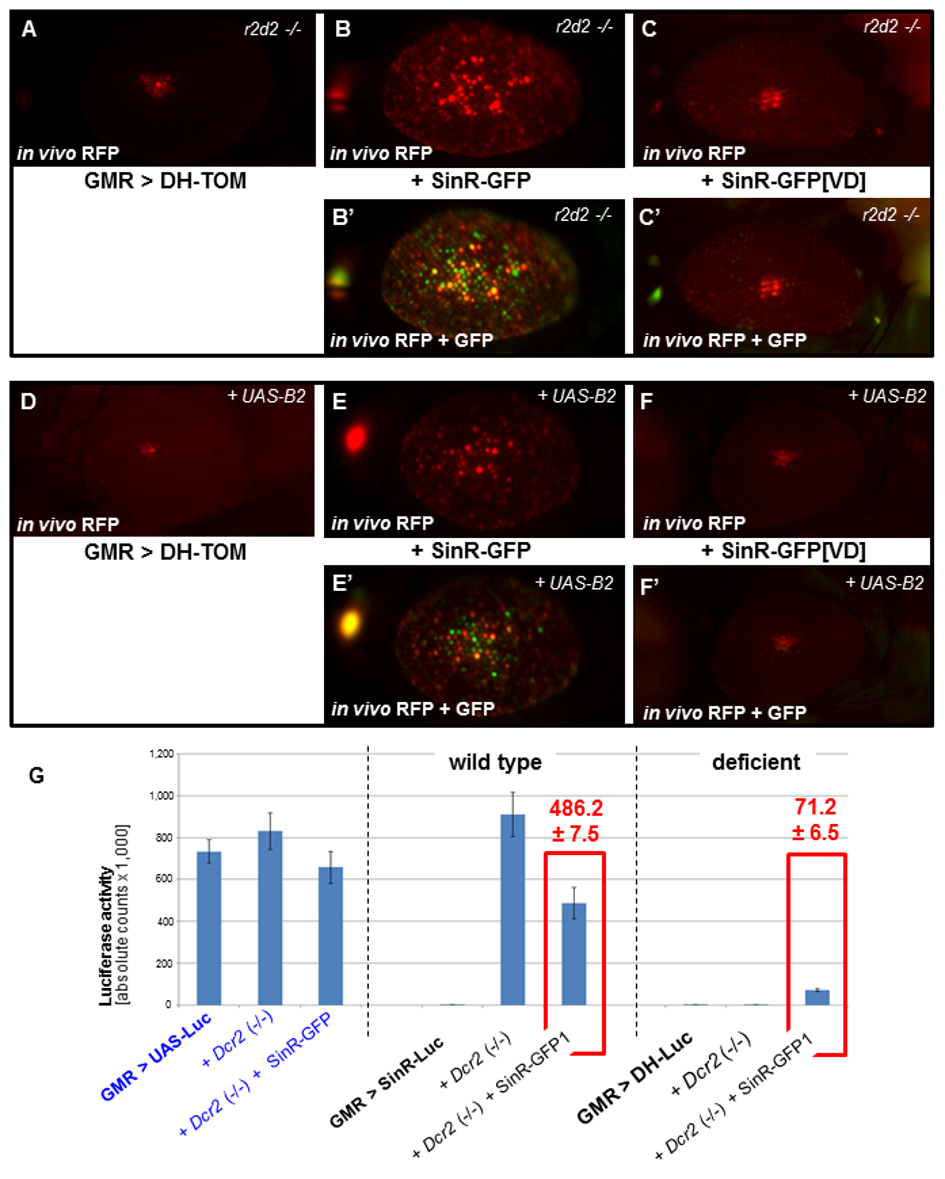

Supplement: Figure S4 — Trans-activation of defective reporter replicons. A. Expression of the DH-TOM defective replicon in the adult eye, when driven with GMR-GAL4 in homozygous r2d2 mutants. Weak expression is seen in the ‘deep pseudopupil’. B. Strong levels of myr:Tomato expression from the defective replicon activated in trans, from a 2nd GFP-expressing replicon (SinR-GFP) contributing a wild type RdRP in trans, in r2d2 mutants. C. Under the same conditions, the point-mutated, replication-deficient replicon SinR-GFP[GVD] fails to activate Tomato expression in trans. D–F. Same experiments as above, using UAS-B2 over-expression to inactivate the RNAi pathway. G. Direct comparison of Luciferase activity levels (Luminometer counts per µL homogenate, per fly), of GMR-GAL4 driving expression of UAS-Luc, SinR-Luc, and DH-Luc in wild type flies, in Dcr2 homozygotes, as well as when the GFP replicon SinR-GFP is co-expressed in Dcr2 mutants (red boxes). All numbers were re-plotted from previous graphs, for better comparison. Note that trans-activation of DH-Luc results in very low activity levels. (TIF) [file pone.0112092.s004.tif]

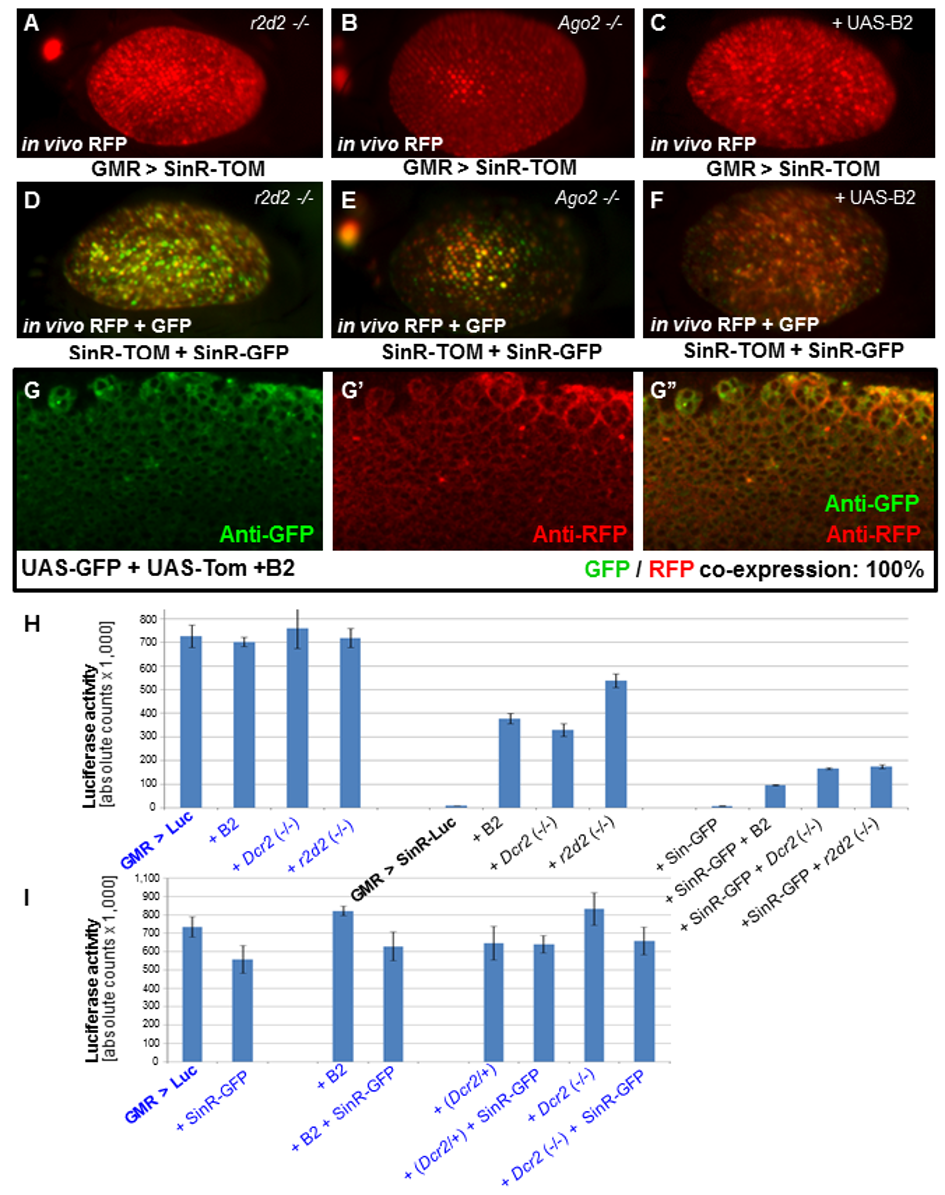

Supplement: Figure S5 — Superinfection exclusion of Sindbis particles. A–C. Additional genotypes to block the RNAi pathway and enable strong levels of SinR-TOM expression in the adult eye, using GMR-GAL4 (from left: r2d2 homozygotes (A), Ago2 homozygotes (B), and UAS-B2 co-expression (C). D–F. Same genotypes co-expressing two replication-competent replicons, SinR-GFP and SinR-TOM, using GMR-GAL4. Many ommatidia choose expression of one replicon over the other (+UAS-B2 eyes were rough and therefore more difficult to analyze). G. Third instar larval eye discs dissected from control flies co-expressing UAS-mCD8GFP and UAS-myr:tdTomato reporter constructs, as well as UAS-B2 under the control of GMR-GAL4. Widespread co-localization of the two fluorescent proteins was observed. H. Exclusion between replicons is independent of the means by which the RNAi pathway is inactivated: the same reduction in Luciferase activity induced by SinR-GFP was observed when co-expressing UAS-B2, in Dcr2 homozygotes, and in r2d2 homozygotes. Activity levels in comparison to UAS-Luciferase are shown for comparison (same data as Figure S1). I. Activity of UAS-Luciferase was not affected by co-expression of Sindbis replicons. Neither co-expression of UAS-B2, nor homozygous (or heterozygous) mutations in Dcr2 affected expression levels, when driven with GMR-GAL4. (TIF) [file pone.0112092.s005.tif]

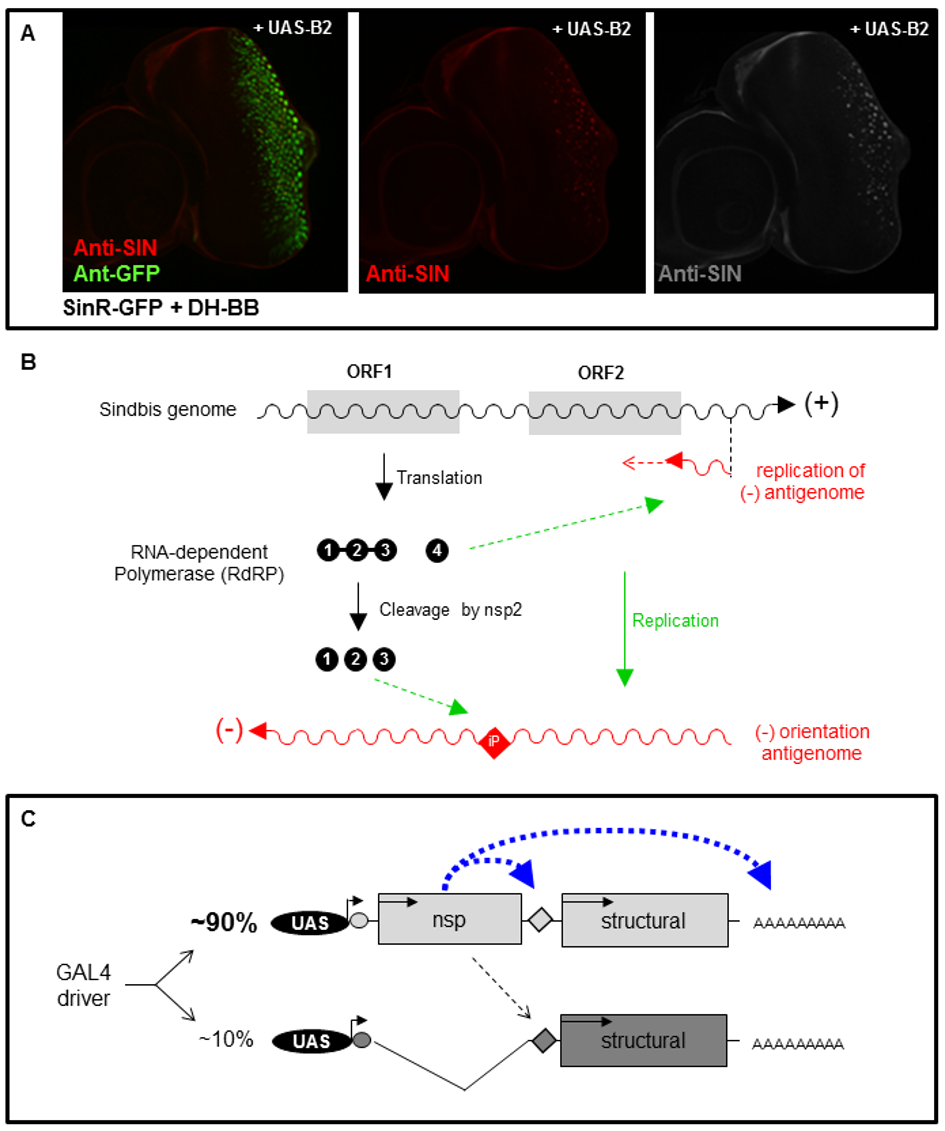

Supplement: Figure S6 — Trans-activation of Sindbis structural proteins and Model. A. Third instar larval eye discs dissected from flies co-expressing SinR-GFP and defective helper DH-BB under the control of GMR-GAL4. Staining with an Antibody against Sindbis (see materials and methods) revealed sparse expression in developing neurons posterior to the morphogenetic furrow (red), where the driver is expressed. B. Summary of RdRP polyprotein cleavage by the nsp2 protease. Note that excess protease activity will abolish RdRP's replication activity, while transcription from the internal promoter on existing antigenomes remains active [30]. C. Additional Model Figure supporting Main Figure 6B, displaying the inability of deficient replicons harboring large deletions spanning ORF1, to induce stochastic exclusion. (TIF) [file pone.0112092.s006.tif]
